# Recurrent Pneumothorax: A Chest Tube Complication

**DOI:** 10.7759/cureus.79236

**Published:** 2025-02-18

**Authors:** Prasansa Basnet, Aayush Gautam, Jenifa Subedi Khatri, Nawaraj Ranabhat, Bigisa Basnet

**Affiliations:** 1 Department of Internal Medicine, Nobel Medical College Teaching Hospital/Kathmandu University, Biratnagar, NPL; 2 Department of Biology, George Mason University, Chantilly, USA; 3 Department of Radiology, Patan Academy of Health Sciences, Patan, NPL; 4 Department of Anaesthesiology, Nepal Armed Police Force Hospital, Kathmandu, NPL

**Keywords:** adolescents, chest tube, chest tube drainage system, kinking of a chest tube, recurrent pneumothorax

## Abstract

Chest tubes are widely used for the management of primary spontaneous pneumothorax. The most common complication of the chest tube is a nonfunctioning chest tube, which might lead to recurrent pneumothorax during hospital admission. We describe a case of a 13-year-old tall and thin-built African American male who presented to the emergency department (ED) with a large right-sided pneumothorax diagnosed with a chest X-ray (CXR). Pneumothorax was managed with the three-compartment chest tube drainage system. Serial CXR revealed improvement in pneumothorax after the chest tube insertion. The patient had recurrent pneumothorax on the right side on the second day of admission attributed to the kinking of a chest tube. Relieving the kink led to the resolution of pneumothorax. The provider needs to be aware of the common complications of chest tube insertion and their management. Alternative treatment options with fewer complications like needle tube insertion for the management of primary spontaneous pneumothorax in children and adolescents need to be considered.

## Introduction

Pneumothorax is the abnormal collection of air in the pleural space between the lung and the chest wall. Pneumothorax is broadly classified into spontaneous and traumatic. Spontaneous pneumothorax is further classified into primary, which occurs in the absence of underlying lung disease, and secondary, which occurs in the presence of existing lung disease, respectively [1,2].

The most common risk factors associated with spontaneous pneumothorax are young age, male sex, thin stature, and tobacco smoking. The high-risk age group for primary spontaneous pneumothorax ranges from 13 to 22 years of age [1,3]. The risk of recurrence after a spontaneous pneumothorax is high in adolescents [4].

Chest tubes are lifesaving instruments in pneumothorax. One of the most common complications associated with the chest tube is recurrent pneumothorax [5]. Although there are studies on recurrent pneumothorax in children and adolescents, studies on recurrent pneumothorax due to chest tube complications in children and adolescents are sparse. Here, we describe a 13-year-old boy with recurrent pneumothorax associated with the kinking of a chest tube.

## Case presentation

A 13-year-old African American male presented to the emergency department (ED) at Nobel Hospital in Biratnagar, Nepal, with a sudden onset of central chest pain for one hour while he was resting on his couch watching television. He reported acute onset continuous chest pain with no radiation, which was made worse with inspiration and movement. He denied cough, fever, recent respiratory tract infection, recent trauma, or similar episodes in the past. He had no pertinent past medical or surgical history, history of vision changes, heart disease, or joint problems. He denied a family history of heart disease, Marfan syndrome, or pulmonary disease. He liked playing basketball, and he denied taking any medications, cigarette smoking, alcohol consumption, or substance use.

A physical examination revealed a height and weight of 169 cm (95th percentile) and 41.2 kg (25th percentile), respectively. His calculated body mass index (BMI) had an underweight BMI of 14.4 kg/m^2^. His vital signs were notable for a temperature of 98°F, heart rate of 127, blood pressure of 115/60 mmHg, respiratory rate of 20, and oxygen saturation of 94% in room air. Trachea was midline. A chest wall exam revealed no reproducible tenderness on palpation. Chest auscultation was notable for decreased breath sounds on the right, with hyperresonant lung fields on the right side and normal bilateral vesicular breath sounds. Heart sounds were normal, with no murmurs or gallops. A neurological examination revealed he was alert and oriented to time, place, and person. The remainder of the neurological exam was non-focal. Laboratory investigations were unremarkable, as indicated in Table 1 and Table 2.

**Table 1:** Complete blood count and absolute differentials for the patient on admission

Test	Patient’s Result	Reference Range
White blood cell count (k/mcL)	10.30	4.5-13.5
Platelet count (k/mcL)	214	150-450
Red blood cell count (M/mcL)	5.28	4.1-5.6
Hemoglobin (g/dL)	15.2	12.5-16.1
Hematocrit (%)	45.2	37-49
Mean corpuscular volume (fL)	85.5	77-95
Red cell distribution width (%)	12.9	11.5-14.5
Neutrophils (k/mcL)	4.68	1.5-8.0
Eosinophils (k/mcL)	0.10	0.05-0.5
Basophils (k/mcL)	0.00	0.0-0.2
Monocytes (k/mcL)	0.90	0.2-1.0
Lymphocytes (k/mcL)	2.50	1.5-7.0

**Table 2:** Blood gas, metabolic parameters, and coagulation profile for the patient on admission BUN = blood urea nitrogen, INR = international normalized ratio, pCO2 = partial pressure of carbon dioxide, pH = potential of hydrogen, PT = prothrombin time, PTT = partial thromboplastin time

Test	Patient’s Result	Reference Range
pH, venous	7.35	7.31-7.41
pCO2, venous (mmHg)	50	38-50
Sodium (mmol/L)	141	135-145
Potassium (mmol/L)	4	3.5-5.1
Chloride (mmol/L)	100	98-107
Carbon dioxide (mmol/L)	29	22-28
BUN (mg/dL)	12	7-20
Creatinine (mg/dL)	0.74	0.5-1.0
Glucose (mg/dL)	95	70-100
Albumin (g/dL)	4.8	3.5-5.2
Bilirubin (total) (mg/dL)	0.6	0.1-1.0
Total protein (g/dL)	7.4	6.4-8.3
Alanine aminotransferase (U/L)	25	8-40
Aspartate aminotransferase (U/L)	24	10-40
Alkaline phosphatase (U/L)	160	44-147
PT (seconds)	12	11-13.5
INR	1.0	0.8-1.2
PTT (seconds)	26	25-35

**Figure 1:**
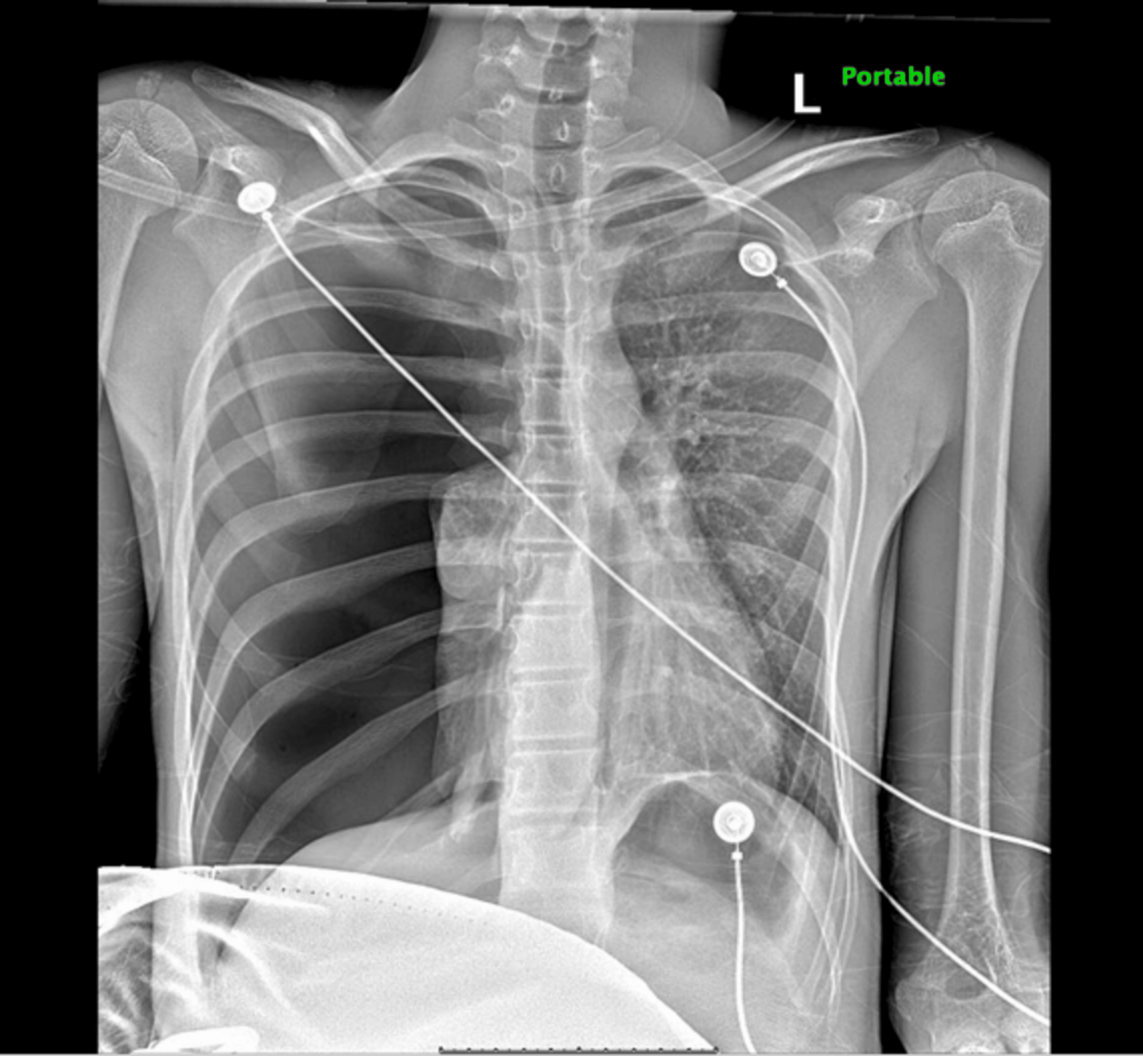
Chest X-ray demonstrating right-sided pneumothorax with associated right lung collapse

A chest X-ray (CXR) obtained in the ED demonstrated a large right-sided pneumothorax (Figure 1).

**Figure 2:**
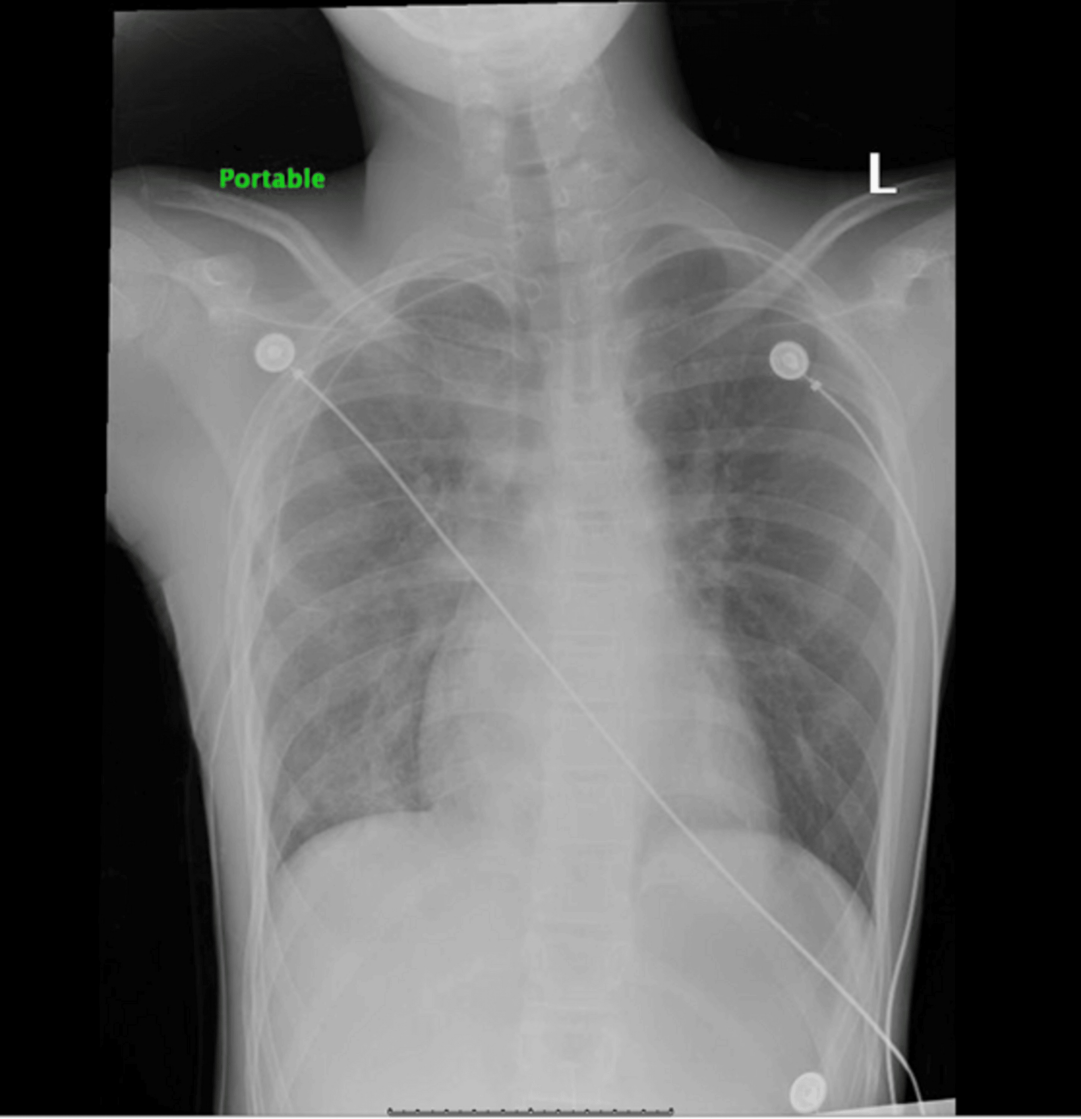
Resolution of right-sided pneumothorax with expansion of the right lung and chest tube placement on the right

He was started on oxygen supplementation via a non-rebreather mask at 5 L/minute. A 28 Fr chest tube was inserted at the right fifth intercostal space and placed on suction, resulting in the improvement of symptoms. A CXR in the ED after the insertion of a chest tube showed improvement (Figure 2), and our patient was admitted to the pediatric floor for further management.

**Figure 3:**
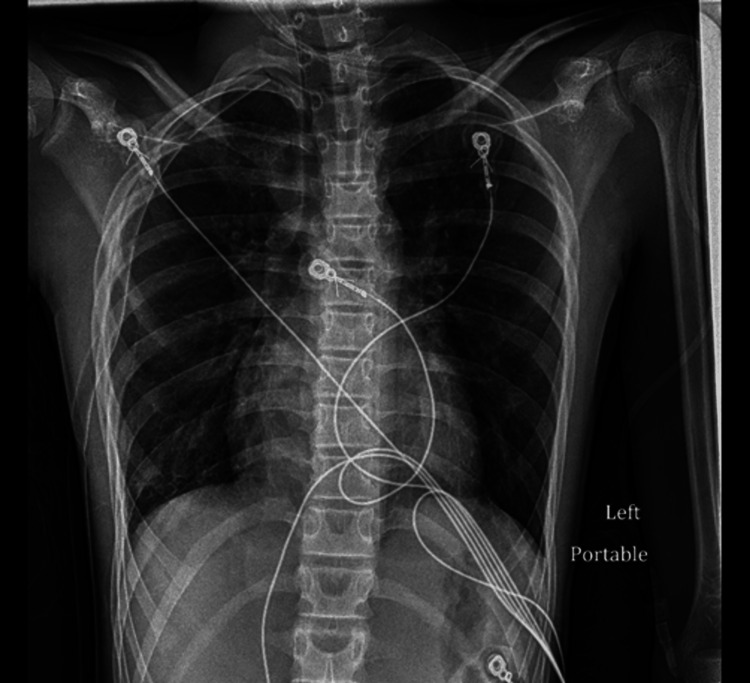
Resolution of right-sided pneumothorax with expansion of the right lung and chest tube placement on the right

During admission, he received acetaminophen 650 mg every six hours and ketorolac 15 mg every six hours as needed for pain management. The three-compartment chest tube drainage system was placed below the chest level of the patient and connected to wall suction at -80 cm H2O. The chest tube drainage system suction pressure was set to -20 cm H2O with adequate expansion of the orange bellow indicator. The chest tube dressing was noted to be dry and occlusive, with no subcutaneous emphysema. Intermittent bubbling in the water seal chamber and good tidaling were noted on the first day of admission. A repeat CXR six hours after admission to the pediatric floor revealed improvement of the pneumothorax with re-expansion of the lung (Figure 3).

**Figure 4:**
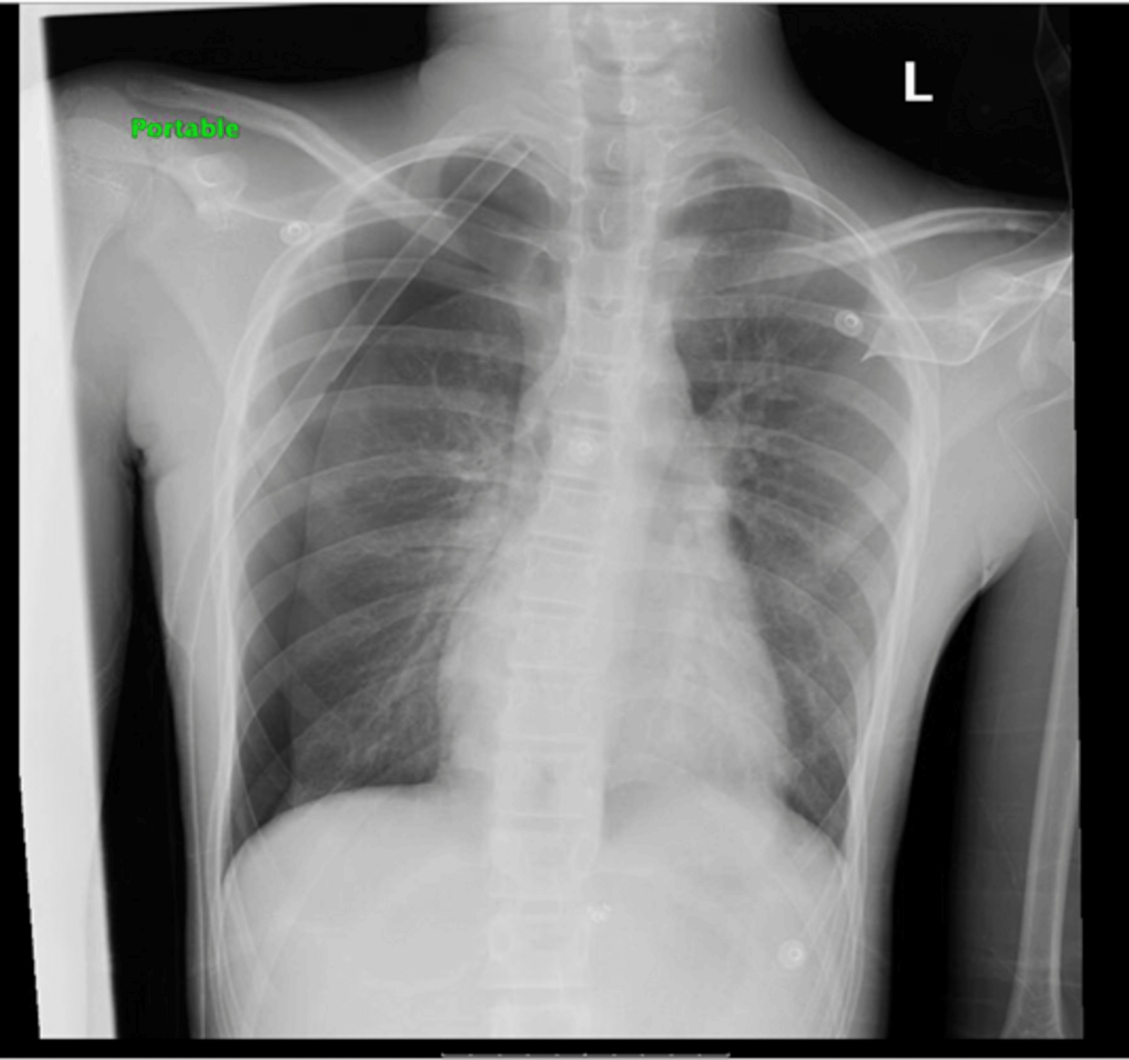
Recurrent 50% right-sided pneumothorax with collapse of the right lung and chest tube placement on the right

On the second day of admission, the patient complained of increasing pain on the right side of his chest. He was hemodynamically stable. The chest tube insertion site was unremarkable. A minimal air leak and no tidaling were observed in the chest tube drainage system. A portable CXR was repeated at the bedside and was notable for recurrent pneumothorax with the collapse of the right lung despite the chest tube being in place (Figure 4).

**Figure 5:**
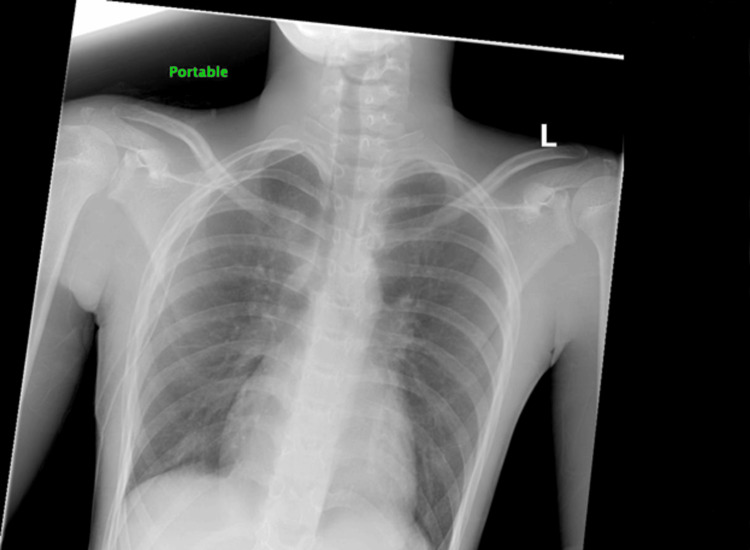
No residual or recurrent pneumothorax with chest tube on the right

The tube was noted to be kinked during observation of the tubing system while the patient was lying in bed. Specifically, the kink occurred at the point where the tube was positioned between the mattress and the patient’s body, leading to partial obstruction. Upon repositioning the tube and relieving the kink, bubbling was noted in the water seal chamber with good tidaling. The patient reported improvement of the chest pain; a CXR was repeated six hours after relieving the kink, which revealed improvement with re-expansion of the lungs (Figure 5).

**Figure 6:**
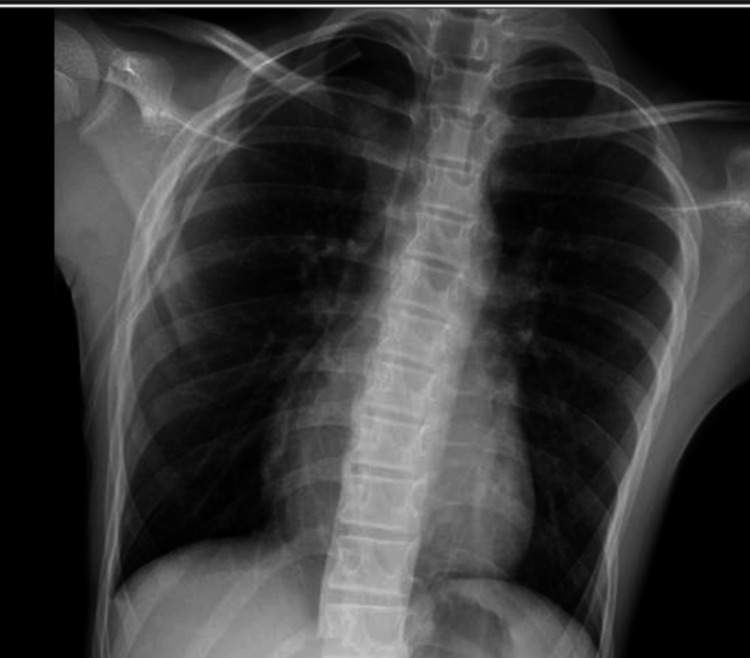
No residual or recurrent pneumothorax with chest tube on the right

The wall suction was discontinued on the sixth day of admission since the patient remained clinically stable, and minimal air leak was noted in the water seal chamber. A CXR after discontinuation of wall suction was noted to be unremarkable (Figure 6).

**Figure 7:**
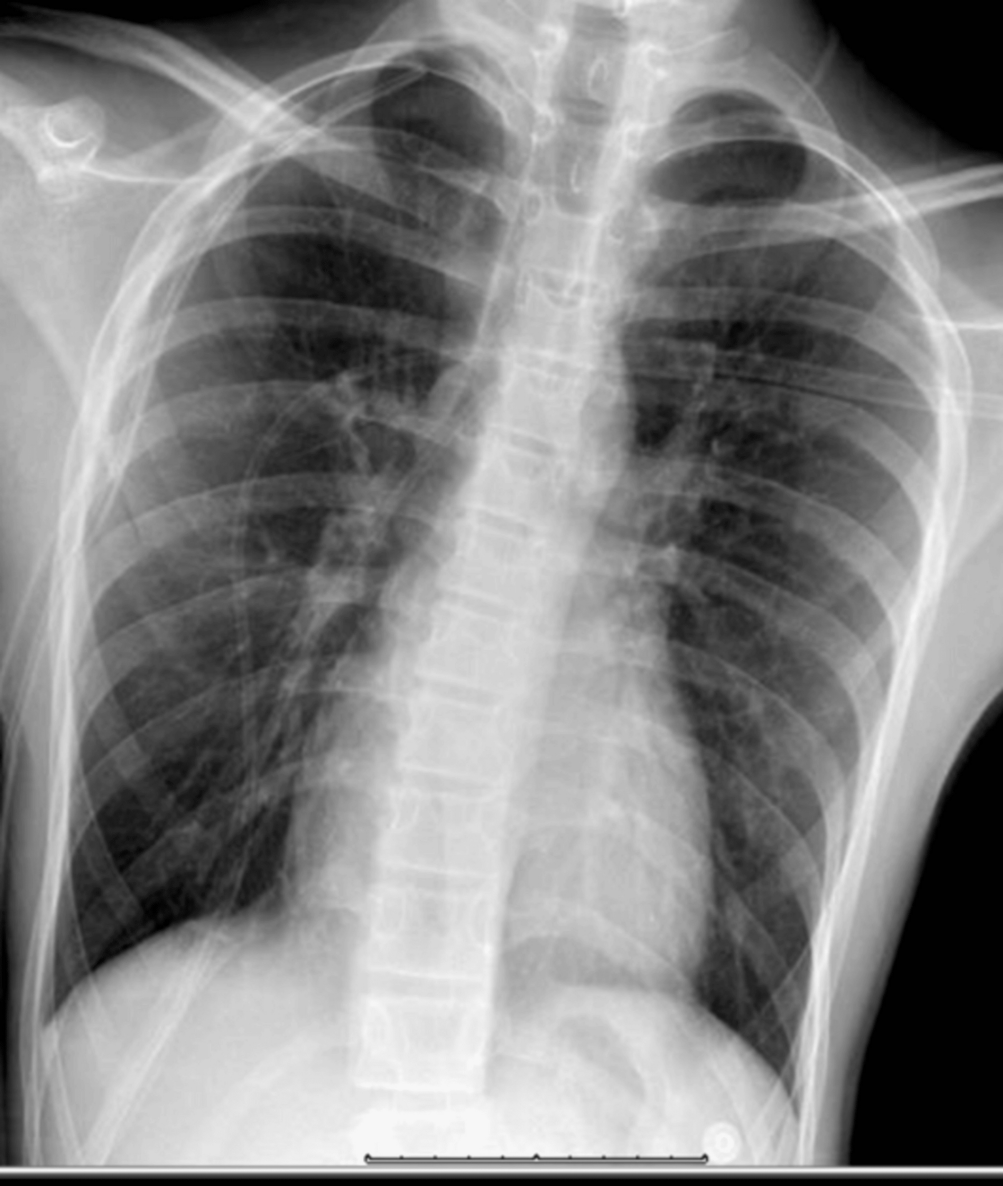
No residual or recurrent pneumothorax with a chest tube on the right

Once no bubbling was noted in the water seal, the chest tube was clamped on the eighth day of admission, and the patient was observed. A CXR five hours later revealed no residual pneumothorax (Figure 7), and the chest tube was removed. He was scheduled for a follow-up CXR two weeks after discharge.

## Discussion

We present a case of a 13-year-old male who had recurrent pneumothorax during hospital admission. Young age, male sex, low BMI, and larger pneumothorax at initial presentation are risk factors for recurrent pneumothorax present in our patient [3,4]. The recurrent pneumothorax was attributed to the kinking of a chest tube because repositioning the chest tube and relieving the kink resulted in the resolution of pneumothorax. To the best of our knowledge, this is the first case report of recurrent pneumothorax in an adolescent due to kinking of a chest tube.

The treatment of primary spontaneous pneumothorax depends upon the size of the pneumothorax. Small-sized primary spontaneous pneumothorax (defined as <50% volume of the hemithorax) is managed with observation and oxygen supplementation as needed [1,2,5]. There are different recommendations for the initial management of large-sized primary spontaneous pneumothorax. The British Thoracic Society (BTS) guidelines recommend needle aspiration, whereas the American College of Chest Physicians recommends a chest tube over needle aspiration for the management of large-sized primary spontaneous pneumothorax [6].

Chest tube drainage is a more invasive procedure compared to needle aspiration. The advantages of needle aspiration are shorter hospital stays, less painful procedures, and lower complication rates compared to chest tubes [5,6]. Studies to evaluate the effectiveness of needle aspiration over chest tubes for the initial management of primary spontaneous pneumothorax in children and adolescents are lacking. Common complications associated with chest tube insertion are local infection at the insertion site and nonfunctioning chest tubes. Disconnection of the chest tube from the drainage system, dysfunctional suction mechanism, tube obstruction due to blood clots, tissue debris, or kinking of the chest tube are commonly associated with nonfunctioning chest tubes. A nonfunctioning chest tube might result in the buildup of negative pressure in the pleural cavity, which might result in recurrent pneumothorax in hospital settings [5-10]. The recurrent pneumothorax attributed to the kinking of a chest tube in our patient could have been possibly avoided using needle aspiration for the management of pneumothorax. Future studies directed toward comparing needle aspiration versus chest tubes in adolescents for the management of primary spontaneous pneumothorax are needed.

The three-compartment chest tube drainage system used in our patient is the most used chest tube drainage system for the management of pneumothorax. It is a complex system that requires proper knowledge, experience, and close observation. The three chambers include the collection chamber, water seal chamber, and suction control chamber, which are interconnected. The collection chamber collects air. The water seal chamber holds a 2 cm column of water, which prevents the air from going back into the pleural cavity during inspiration. Bubbling is initially observed in the water seal chamber during the management of pneumothorax, which decreases over time with the resolution of pneumothorax. Continuous and vigorous bubbling indicates a persistent air leak, which needs further evaluation. Tidaling is also observed in the water seal chamber, which is the oscillation of the water level during respiratory cycles. Tidaling is poor or absent if the chest tube is occluded or kinked [8,11]. In our patient, the tidaling was noted to be absent on the second day of admission due to kinking of the chest tube. Tidaling might also be absent in a fully expanded lung. Hence, tidaling should be correlated with the clinical status of the patient and the air leak in the water seal chamber. The suction chamber has the suction level adjusted to -20 cm H2O. The suction chamber is either attached to continuous wall suction normally adjusted to -80 cm H2O, or it can be placed underwater seal drainage with no wall suction mechanism, or it can be initially attached to wall suction and then transitioned to water seal drainage with the discontinuation of wall suction as observed in our patient. Although chest tubes can be lifesaving instruments, proper evaluation of the chest tube drainage system after its placement is very important. The operator should be familiar with the chest tube drainage system so that they can think through the differential diagnoses in case of nonfunctioning chest tubes and address the potential complications, which ultimately lead to improved patient outcomes.

## Conclusions

Our report describes a 13-year-old, tall, and thin-built male who had recurrent primary spontaneous pneumothorax during hospital admission due to the kinking of a chest tube. Although the chest tube drainage system is widely used for the management of large-sized primary spontaneous pneumothorax in children and adolescents, it is associated with various complications, including dislodgement, kinking, and mechanical obstruction. Our report aims to highlight a crucial learning point for clinicians to always consider and reassess simple mechanical causes before pursuing more complex evaluations. The operator should have good knowledge and experience regarding the evaluation and management of complications of the chest tube drainage system. Alternative treatment options like needle aspiration need to be considered for the management of primary spontaneous pneumothorax in children and adolescents due to their lower complication rates.
